# Citric Acid-Mediated Abiotic Stress Tolerance in Plants

**DOI:** 10.3390/ijms22137235

**Published:** 2021-07-05

**Authors:** Md. Tahjib-Ul-Arif, Mst. Ishrat Zahan, Md. Masudul Karim, Shahin Imran, Charles T. Hunter, Md. Saiful Islam, Md. Ashik Mia, Md. Abdul Hannan, Mohammad Saidur Rhaman, Md. Afzal Hossain, Marian Brestic, Milan Skalicky, Yoshiyuki Murata

**Affiliations:** 1 Graduate School of Environmental and Life Science, Okayama University, Okayama 700-8530, Japan; muta@cc.okayama-u.ac.jp; 2 Department of Biochemistry and Molecular Biology, Bangladesh Agricultural University, Mymensingh 2202, Bangladesh; hannanbmb@bau.edu.bd (M.A.H.); mafzalbau@bau.edu.bd (M.A.H.); 3 Plant Breeding Division, Bangladesh Rice Research Institute, Gazipur 1701, Bangladesh; ishrat.bau@gmail.com; 4 Department of Crop Botany, Bangladesh Agricultural University, Mymensingh 2202, Bangladesh; masudcbot@bau.edu.bd (M.M.K.); ashik41570@bau.edu.bd (M.A.M.); 5 Department of Agronomy, Khulna Agricultural University, Khulna 9100, Bangladesh; shahin.imran@kau.edu.bd; 6 Chemistry Research Unit, United States Department of Agriculture—Agricultural Research Service, Gainesville, FL 32608, USA; charles.hunter@usda.gov; 7 Department of Fisheries, Bangamata Sheikh Fojilatunnesa Mujib Science and Technology University, Melandah, Jamalpur 2012, Bangladesh; saiful@bsfmstu.ac.bd; 8 Department of Seed Science and Technology, Bangladesh Agricultural University, Mymensingh 2202, Bangladesh; saidursst@bau.edu.bd; 9 Department of Plant Physiology, Slovak University of Agriculture, 94976 Nitra, Slovakia; marian.brestic@uniag.sk; 10 Department of Botany and Plant Physiology, Faculty of Agrobiology, Food and Natural Resources, Czech University of Life Sciences Prague, 16500 Prague, Czech Republic; skalicky@af.czu.cz

**Keywords:** citrate, heavy metal stress, drought stress, antioxidant, reactive oxygen species, salinity, aluminum toxicity

## Abstract

Several recent studies have shown that citric acid/citrate (CA) can confer abiotic stress tolerance to plants. Exogenous CA application leads to improved growth and yield in crop plants under various abiotic stress conditions. Improved physiological outcomes are associated with higher photosynthetic rates, reduced reactive oxygen species, and better osmoregulation. Application of CA also induces antioxidant defense systems, promotes increased chlorophyll content, and affects secondary metabolism to limit plant growth restrictions under stress. In particular, CA has a major impact on relieving heavy metal stress by promoting precipitation, chelation, and sequestration of metal ions. This review summarizes the mechanisms that mediate CA-regulated changes in plants, primarily CA’s involvement in the control of physiological and molecular processes in plants under abiotic stress conditions. We also review genetic engineering strategies for CA-mediated abiotic stress tolerance. Finally, we propose a model to explain how CA’s position in complex metabolic networks involving the biosynthesis of phytohormones, amino acids, signaling molecules, and other secondary metabolites could explain some of its abiotic stress-ameliorating properties. This review summarizes our current understanding of CA-mediated abiotic stress tolerance and highlights areas where additional research is needed.

## 1. Introduction

Abiotic stresses such as drought, flooding, high temperature, low temperature, salinity, and heavy metals (HM) inhibit plant growth and lower yield potentialities in crops [1]. As climate change leads to less predictable and more extreme weather events, environmental stresses have become a major threat to food security [2]. About 90% of arable lands are prone to one or more environmental stress [3] and abiotic stresses already account for up to 50% yield loss in many major crops [1]. Abiotic stresses cause changes in plant metabolism, growth, and development and in extreme cases lead to plant death [4,5]. The exogenous application of protective plant metabolites like citric acid or citrate (CA) has emerged as an effective approach to improve plant resilience to environmental stresses and thus sustain food production.

**Figure 1:**
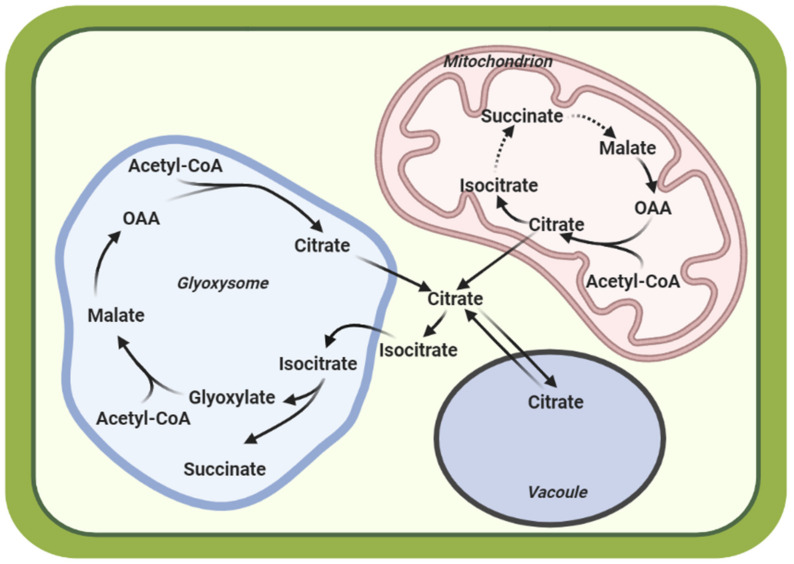
A simplified model showing the biosynthetic pathway of CA in plant cells. CA biosynthesis occurs in the TCA cycle in the mitochondria or via the Glyoxylate cycle in the glyoxysome. CA is exported to the cytosol where it can remain or be stored in the vacuole. Citric acid/Citrate, CA; oxaloacetate, OAA.

Citric acid, a 6-carbon tricarboxylic acid synthesized by the citrate synthase (CS)-catalyzed condensation of oxaloacetate (OAA) and acetyl-CoA, is an intermediate of the mitochondrial tricarboxylic acid (TCA) cycle [6,7]. In the glycolytic pathway, glucose is converted to pyruvate, which is transported to the mitochondria and is either oxidized to produce acetyl-CoA or carboxylated to form OAA. Alternatively, OAA can be formed by the catalysis of phosphoenolpyruvate (PEP), an intermediate in glycolysis, by phosphoenolpyruvate carboxylase (PEPC) [8]. OAA serves as a substrate for CA biosynthesis in the TCA cycle [8,9]. In plant cells, CA is also a metabolic intermediate of glyoxylate cycle, which occurs in specialized peroxisomes called glyoxysomes [6] (Figure 1). After being transported into the cytosol, the CA can be utilized by the cell immediately or stored in the vacuole to maintain the cytosolic pH [9,10] (Figure 1).

More than a decade ago, it was reported that plants growing in alkaline soils exude CA and malate from their roots and that this enables them to uptake essential nutrients like phosphorus and iron by decreasing the pH of the rhizosphere [11]. Since then, several studies have demonstrated that the positive effects of CA are not from the pH modulation along, but that there are also many physiological responses by plants to exogenously applied CA. In addition, application of CA improved physiological parameters in numerous plant species such as *Polianthes tuberosa* [12], *Lilium spp.* [13], and *Phaseolus vulgaris* (common bean) [14]. Moreover, CA has also been used to mitigate drought, salinity, temperature, and HM stresses in a variety of plant species [15,16,17,18]. This review discusses the current understanding of the physiological and biological roles of CA in enhancing abiotic stress tolerance to salinity, drought, HMs, alkalinity, and temperature.

## 2. Effects of Abiotic Stress on Endogenous CA Levels

Abiotic stresses trigger complex responses in plants involving diverse signaling events, physiological adjustments and activation of defense mechanisms that together result in changes to the biosynthesis, transport, and storage of many primary and secondary metabolites (SMs). Various types of experimental evidence have demonstrated that abiotic stresses can influence endogenous CA levels in plants (Table 1). In some plant species, such as *Helianthus annuus* (sunflower), *Solanum lycopersicum* (tomato), *Acacia ampliceps*, and *Trigonella foenum-graecum*, CA increased after 7 days to 4 weeks of salinity exposure [19,20,21,22]. Tomato, *Gossypium hirsutum*, *Clusia* sp. and *Aptenia cordifolia*, showed large increases in endogenous CA levels under drought stress [23,24,25,26], whereas levels did not change in *Solanum tuberosum* (potato) [27]. In *Festuca arundinacea*, hybrid bermudagrass and *Lolium arundinaceum*, endogenous CA levels increased under heat stress [17,28,29], whereas no change in CA was observed in the tuber or leaf of potato nor in the leaf of *Poa pratensis* [26,28]. 

Various studies have reported that endogenous CA accumulates after exposure to HM stresses. Exposure to cadmium (Cd) or nickel (Ni) causes CA accumulation in the roots of *Solanum nigrum*, the shoots of *Brassica juncea* and *Sesuvium portulacastrum* and both the roots and shoots of *Amaranthus paniculatus*, while causing a CA decrease in roots of *Sesuvium portulacastrum* [30,31,32,33]. Another study showed that endogenous CA levels increased in *Oryza sativa* (rice) after exposure to 50 µM chromium (Cr) for 8 days [34]. A large increase in endogenous CA levels in root exudates from *Secale cereale*, *Triticum aestivum* (wheat), *Glycine max* (soybean), rice, *Zea mays* (maize), *Pisum sativum* (pea), *Hordeum vulgare* (barley), and *Cassia tora* has been observed under aluminum (Al) stress [35,36,37,38,39].

In general, endogenous CA levels tend to increase in response to salinity, drought, heat, and HM stresses. The degree and longevity of increased CA are specific to the plant species and the type of abiotic stress. Exogenous application of CA appears to improve the tolerance of plants to abiotic stress [17,18,40,41]. Despite the growing number of scientific investigations, our understanding of the effects endogenous CA or exogenously applied CA on abiotic stress tolerance in plants remains limited. In the following sections, we describe the roles of exogenous CA application in ameliorating plant stress responses to various abiotic stresses. 

## 3. Exogenous CA for Mitigation of Abiotic Stress

### 3.1. Salinity Stress

Exogenous application of CA can increase the salinity tolerance of plants and ultimately increase growth and yield (Table 2). *Carica papaya* (papaya) seeds primed with a CA solution showed improved germination under salt stress conditions [42]. A foliar spray with CA reduced the sensitivity of *G. barbadense* to salt stress, improving growth and yield, and led to higher total soluble sugars (TSS), total soluble protein (TSP), total phenolic compounds (TPCs), free amino acids (FAA), and proline content [18]. Moreover, El-Hawary and Nashed [43] reported that the foliar application of CA in combination with ascorbic or salicylic acid enhanced the growth and productivity of maize under salt stress conditions. Application of CA in combination with ascorbic acid and thiamin improved salinity tolerance by upregulating the non-enzymatic antioxidants (TPCs and proline accumulation) and decreasing enzymatic antioxidants [CAT, POX, and phenylalanine ammonia lyase (PAL)] in *H. sabdariffa* and *Melissa officinalis* (lemon balm), a response related to the maintenance of the cellular redox state [44,45]. Several studies have shown that CA application can increase the activity of antioxidants, including superoxide dismutase (SOD), peroxidase (POX), catalase (CAT), glutathione peroxidase (GPX), polyphenol oxidase (PPO), and ascorbate peroxidase (APX) in cotton, maize, *Beta vulgaris* (sugar beet), *Hibiscus sabdariffa*, and *Leymus chinensis* (Chinese ryegrass) [18,43,45,46,47]. Essential oil components (monoterpene hydrocarbons and oxygenated sesquiterpenes) of lemon balm under salt stress conditions were increased by CA treatment [44]. The application of CA in sugar beet individually or in combination with peel extracts of banana and/or tomato improved tap roots yield in saline soil [47].

### 3.2. Drought Stress 

Application of exogenous CA improved drought tolerance and increased productivity of *Lilium Cv. Brunello* and cotton plants [13,15]. Accumulation of some OAs including CA is associated with improved drought tolerance in *G. barbadense* [48]. Exogenous application of CA improved the growth of *Brassica oleracea var. capitata* (cabbage) seedlings in drought-affected areas by alleviating oxidative stress [49]. Additionally, in cabbage, CA was shown to increase phosphorus uptake and decrease hydrogen peroxide (H_2_O_2_) accumulation [49]. In common bean plants, exogenous application of CA increased the relative water content (RWC) and chlorophyll (Chl) content of leaves, leading to increased growth and productivity [14]. Several morphological and yield-related traits, metabolite concentrations, (Chl *a*, Chl *b*, Chl *a*+*b*, carotenoid, and proline), and antioxidant enzyme activities (CAT, POX, and APX) were increased by exogenously applied CA in *Gossypium barbadense* [15].

### 3.3. Temperature Stress

High temperature stress results in decreased yield in many crops. However, it has been reported that exogenous CA can alleviate heat stress in several plant species (Table 3). Spraying of 20 mM CA on leaves of *Lolium arundicaceum* significantly improved photosynthetic efficiency, Chl biosynthesis, and activity of antioxidant enzyme such as SOD, POX, and CAT. The enhanced antioxidant system alleviated cell membrane damage (lower electrolyte leakage (EL) and malondialdehyde (MDA) content), ROS accumulation, and leaf senescence [17]. In addition, foliar spraying of CA application at the rate of 2.5 and 5 g L^−1^ increased fruit setting and yield in tomato under heat stress [50]. There has also been at least one report of CA application alleviating low temperature stress, as CA application suppressed defoliation and increased leaf number in *Hibiscus rosa-sinensis* under cold stress treatment [51].

### 3.4. Alkalinity Stress

It has been also reported that application of CA can ameliorate the effects of alkalinity stress. Treatment of Chinese ryegrass with 50 mg L^−1^ CA increased alkaline stress tolerance, improving growth, relative growth rate, photosynthesis and activities CAT, SOD, and APX in plants grown in the presence of 100 mM Na_2_CO_3_ [46]. Another study showed that soil treatment with CA at 40, 80, and 120 mg kg^−1^ significantly improved plant growth features such as plant biomass, root development, root–shoot ratio, and total root surface area and also increased soil nutrients of *Rosa roxburghii* seedlings under alkaline stress [52].

### 3.5. Heavy Metal Stress

Exposure to HMs causes plant stress and reduces plant growth and biomass production. Application of exogenous CA has been shown to mitigate HM stress in numerous instances (Table 4). Plants treated with CA had improved growth and biomass accumulation, increased photosynthesis and Chl content, higher water use efficiency, and higher antioxidant enzymes activity, and reduced ROS, MDA, and EL [53,54,55]. 

Exogenous CA (2.5 mM) in the growth medium of Cu-stressed (100 µM) *Brassica napus* increased shoot and root length, numbers of leaves, and leaf area [56]. Moreover, improved growth and biomass of *B. napus* has been shown for CA-treated plants exposed to Cd stress [16], Pb stress [57], and Cr stress [58]. Similarly, the application of CA at a rate of 100 μmol L^−1^ in nutrient solution reversed the Cd stress-induced loss of root biomass in *Salix variegate* [55].

It is well-known that Chl content is an important contributor to rates of photosynthesis and that exogenous application of CA can mitigate HM stress by increasing Chl content (Table 4). Addition of CA (5 mM) increased total Chl by 18% in Cr-stressed *Helianthus annuus* (sunflower) plants [59], where supplementation with CA was also shown to increase Chl *a*, Chl *b*, and Chl *a+b* contents grown under Cd stress [55], Cr stress [40] and Pb stress [57]. Sebastian and Prasad [60] showed that exogenous CA (50.0 µM) enhanced Cd stress tolerance in rice with increased Chl (54.0–64.0%) and carotenoid (40.0–53.0%) content. Moreover, SPAD values (a measure of Chl content) increased by 35.2% with CA (0.6 mM) foliar application to *B. juncea* under Cd stress [61]. Application of CA also led to a 17% increase in carotenoid content in *Salix variegate* under Cd stress [55] and a 23% increase in sunflower under Cr stress [59]. Kaur et al. [33] reported that soil treatment with 0.6 mM CA ameliorated Cd stress in *B. juncea* with increased total Chl and carotenoid content.

Net photosynthesis rate (Pn) and stomatal conductance (Gs) generally increase in CA-treated plants (Table 4). CA treatment (5 mM) of Cr-stressed (20 mg Cr kg^−1^) sunflower increased Pn, water use efficiency (Pn/E), transpiration rate (E), and Gs by 21%, 53%, 26%, and 12%, respectively [59]. Furthermore, CA increased leaf RWC and reduced the proline content, thereby improving the water status of treated plants [53]. Addition of exogenous CA (2.5 mM) to growth media improved the Pn, E, Gs, and Pn/E of *B. napus* grown under Pb stress (50 and 100 μM) [57]. Both root irrigation and foliar application of CA improved the tolerance to Pb stress in *Larix olgensis*, a response associated with increased proline [54]. Foliar spray of CA (0.6 mM) also increased the proline content (63%) in leaves of *B. juncea* [61]. 

Furthermore, treatment of plants with CA has been shown to reduce HM accumulation (Table 4). Inclusion of exogenous CA (5 mM) in the growth medium led to reduced Cd uptake and mitigated the Cd stress in *Corchorus olitorius* [62]. CA application (0.25 g kg^−1^) decreased Cd uptake by 83.9% in the Sahiwal-2002 maize variety [63]. Another study showed that CA application in conjunction with other OAs such as malic acid or oxalic acid or chelators such as EDTA or DTPA decreased Cd accumulation in wheat, thereby reducing bioavailability of Cd and enhancing tolerance to Cd stress [64]. Sebastian and Prasad [60] reported that addition of CA (50.0 µM) along with malate decreased the Cd translocation (18.0–20.0%) in rice. Similarly, exogenous CA (5.0 mmol L^−1^) reduced Ni uptake by roots in *B. juncea* (leaf mustard) and increased shoot/root ratio (the ratio of shoot to root Ni concentration) and thereby conferred Ni stress tolerance [65].

Production of ROS is a known outcome of HM stress. Addition of exogenous CA dramatically reduced ROS levels and improved stress tolerance in *B. juncea* and *Pisum sativum* [66,67]. Inclusion of CA (2.5 mM) in growth media reduced H_2_O_2_ and MDA contents in both leaves and roots of Pb-stressed *B. napus* [57]. Moreover, Kaur et al. [68] reported that soil containing 0.6 mM CA reduced ROS production and ameliorated Cd stress in *B. juncea*. Foliar spray of CA (0.6 mM) along with SA decreased H_2_O_2_ content by 19% in Cd-stressed *B. juncea* [61]. Kumar et al. [69] reported that exogenous CA (250 μM) in a nutrient solution mitigated Pb stress in tomato, a response associated with decreased α-tocopherol content and MDA levels. Several studies have shown that antioxidant enzyme activities increased with CA treatment and that increased antioxidants improved the stress tolerance in plants (Table 4). Antioxidant enzyme activities such as SOD, POX, CAT, and APX were increased in *B. napus* when the plants were treated with 2.5 mM CA under various HM stress conditions [56,57,58]. Soil treatment with CA (20 mmol kg^−1^) increased antioxidant defense mechanisms and slightly reduced the sensitivity to Cd stress in *Solanum nigram* [70]. Clearly exogenous CA application in plants can help mitigate the effects of HM stress, apparently by improving osmotic balance, HM sequestration, photosynthetic attributes, and antioxidant systems. 

On the contrary, several studies have shown that CA application can enhance uptake of HMs in plants such as Cr in *B. napus* [58] and sunflower [59], Cd in *B. napus* [16], *Solanum nigrum* [70], and *B. juncea* [53], Mn in *Juncus effuses* [71], Pb in *B. napus* [57], and Cu in *B. napus* [56]. Although CA application increased HM uptake, there were no obvious toxicity symptoms associated. Instead, CA treatment helped mitigate HM stress by promoting enhanced growth and biomass, higher Chl content and photosynthesis, higher antioxidant enzyme activity, lower ROS accumulation, and reduced membrane lipid oxidation [55,72], all while accelerating phytoextraction of HMs from the soil [53,56,57,59].

## 4. Mechanisms of CA-Mediated Abiotic Stress Tolerance

### 4.1. Regulation of Heavy Metal Uptake and Sequestration

Plants have developed various strategies to withstand high concentrations of HMs in the rhizosphere, which can impose adverse effects on the growth and physiological processes [73,76]. Selective uptake or efflux of metals at the plasma membrane, chelation of metals in the cytosol by peptides, and compartmentalization of metal ions in the vacuole by tonoplast located transporters are all strategies to limit damage by HMs [76,77].

**Figure 2:**
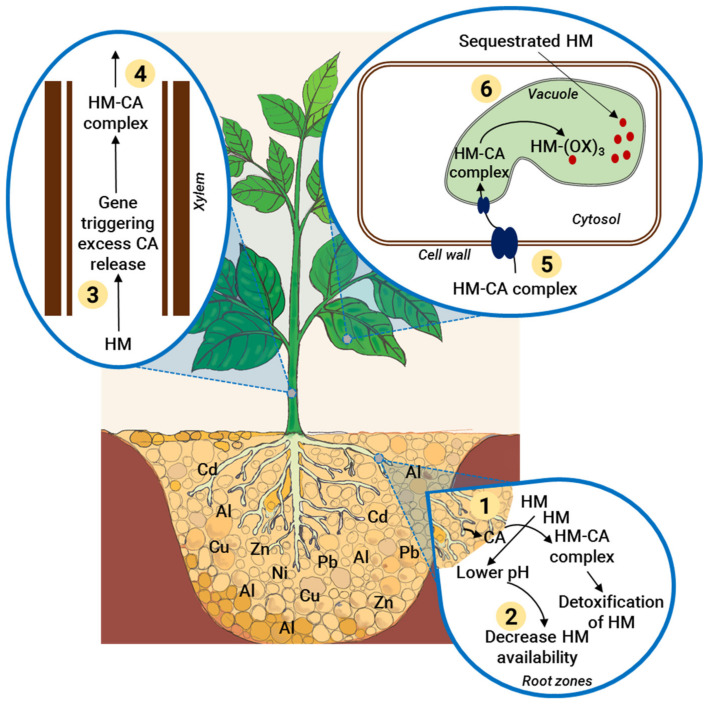
Mechanisms of HM stress tolerance mediated by citric acid (CA). In response to HM-containing soil, (**1**) plant roots release exudate containing CA whereupon CA can detoxify HMs by forming HM-CA complexes. (**2**) Moreover, organic acids like CA decrease the rhizosphere pH and cause precipitation of HMs. (**3**) Sensing of HMs activates genes involved in CA release in the shoot xylem. (**4**) HMs form HM-CA complexes through ligand exchange reactions with citrate. (**5**) Complexes of HM-CA, once transferred from xylem to leaf cells through iron regulated/Ferroportin family transporters or ABC transporters, undergo another ligand exchange reaction to reform HM-oxalate complexes which are deposited in the vacuole. (**6**) HMs are sequestered in the cytosol through phyotochelation and transplanted into tonoplast via transporters.

**Figure 3:**
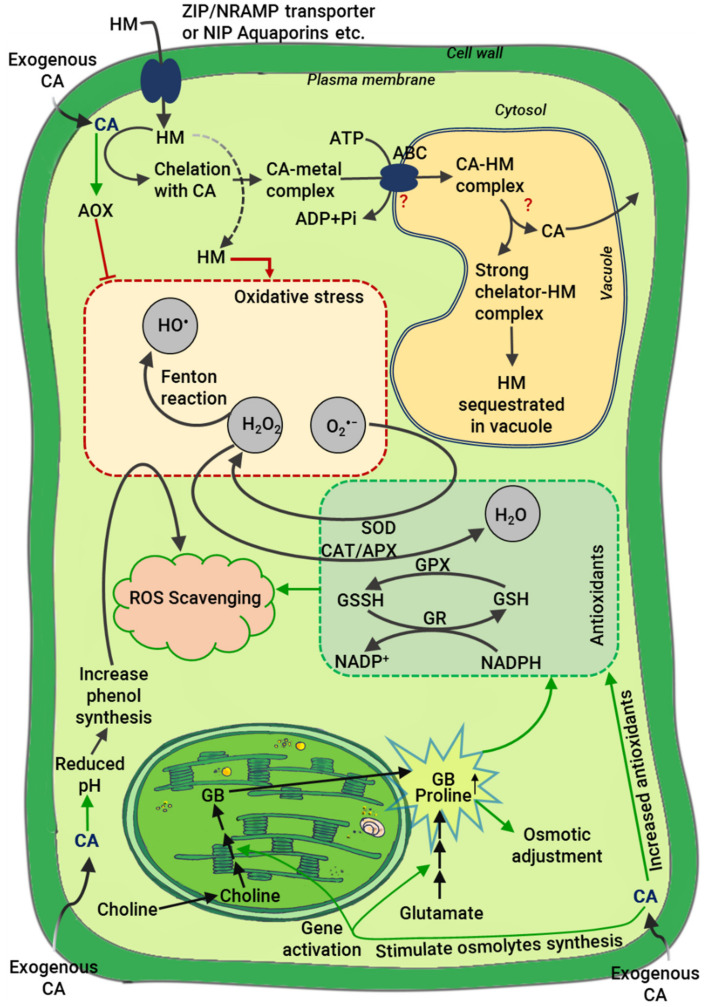
Overview of cellular mechanisms for HM detoxification and stress tolerance involving citric acid (CA). HMs enter cytosol after uptake through anion channels or metal transporters, for example, ZIP (zinc/iron -regulated transporter) family members or NRAMPs (macrophage proteins associated with natural resistance) family members or NIP aquaporin (nodulin-26-like intrinsic proteins of the aquaporin family) etc. Cellular CA functions as high-affinity ligand, chelating HMs in the cytosol and then binding together to form a stable chelation complex via the cytosol ligand exchange reaction. The chelation complex is then transported into the vacuole via vacuolar transporters like ABC (ATP-binding cassette) tonoplast transporter achieving HM sequestration. CA further aids vacuolar compartmentalization or remobilization of HMs by buffering the concentrations of cytosolic HMs, but the precise mechanism remains unclear. HMs induce oxidative stress in cells, leading to the formation of ROS. Exogenous CA enhances antioxidant systems (e.g., glutathione (GSH), superoxide dismutase (SOD), catalase (CAT), glutathione peroxidase (GPX), etc.) to fine-tune ROS levels and maintain normal cellular activities. High cellular CA also activates alternative oxidase (AOX) and that detoxifies ROS. Exogenous CA also induces osmolyte synthesis (e.g., proline, glycine betaine (GB), etc.) which regulates the osmotic balance and promotes ROS scavenging enzyme gene expression. Finally, CA decreases the pH of the cell and increases the synthesis of total polyphenol compounds (TPC) which directly scavenge ROS.

Exogenous application of CA can enhance HM stress tolerance through the detoxification of HMs by chelating them at the root surface, in the xylem, or in the cytosol (Figure 2 and Figure 3). There are a variety of plant-produced high-affinity HM ligands. In the xylem sap, CA is one of the primary ligands for Fe, Cu, Ni, Cd, and Zn [78]. The secretion of OAs, e.g., CA, oxalic acid, malic acid, increases under HM stress [79]. Generally, OAs, including CA, have one or more carboxyl group which acts as a ligand for HMs, chelating HMs and thereby affect their redox behavior by forming non-toxic compounds or preventing their uptake by plant roots [80]. When intercellular HM levels approach toxic levels plants can store them into vacuoles [81]. However, the difference in HM concentration between the vacuolar lumen and the cytosol can be high, presenting the possibility of HM leakage from the vacuole into the cytosol. Most HMs are bound to chelators such as CA inside the vacuole to reduce this risk [82]. After HM chelation in the cytosol or at the root-soil interface, HMs are translocated to the shoot via xylem as non-toxic CA-chelated complexes (Figure 2) [77]. According to Vatansever et al. [83], CA works as a chelator for solubilized Ni, allowing transportation via cation transport systems such as Fe, Mg, Cu, Zn as well as various proteins. Root exudates also have a role in HM tolerance. Root exudates containing high levels of CA make HMs unavailable for plant uptake by forming HM-citrate complexes (Figure 2) [80]. Salt et al. [84] reported that roots of *Thlaspi* sp. secreted Ni-chelating exudates rich in CA and histidine in response to Ni stress, which resulted in decreased Ni uptake. Moreover, Ma and Hiradate [85] showed that CA formed non-toxic Al-citrate complexes in the symplasm of *Hydrangea* grown in the presence of Al. Similarly, buckwheat grown in the presence of Al showed upregulation of genes involved in CA release and increased CA in the xylem, where CA complexes with Al through a ligand exchange reaction [85]. Another study showed that in soybean, Al resistance is promoted exudation of CA by roots [86]. CA has a lower affinity for HMs like Cd, Ni, Co (Cobalt) and Zn and comparatively a strong binding affinity toward Fe and Al [77,87]. The chelating potential and plant growth-promoting role of CA has been reported under various HM stresses, including Cr [58], Cd [16], Pb [57], and Cu [56]. 

The molecular nature of exogenous CA-mediated HM stress tolerance remains poorly understood. In general, a specific tolerance mechanism is adopted by plants for a given HM stress. It is possible that several mechanisms may be involved in reducing the toxicity of HMs. From the above discussion, we can hypothesize exogenous CA application in rooting media may promote HM stress tolerance by directly impairing the uptake of HMs. Moreover, increased intracellular CA accumulation in cells resulting from exogenous application likely improves HM tolerance by acting as an HM chelator, promoting sequestration of HMs into vacuoles (Figure 2 and Figure 3).

Another strategy to limit the uptake of metal ions by plants lies in modifying the rhizosphere pH, which can result in precipitation and insolubility of HMs (Figure 2). One mechanism behind modifying pH involves exudation of OAs like CA [88,89]. Root exudates also serve to concentrate metal ions to the apoplast and help prohibit HMs from entering cellular spaces [90]. Root exudates of several plants including *Secale cereale*, wheat, soybean, rice, maize, pea, and barley grown under Al stress contain high levels of CA [35,36,37,38,39]. 

The central vacuole is the principal metal ion storage compartment in the plant cell [91]. Several families of intracellular transporters located on the tonoplast membrane were identified in plants experiencing HM stress and undergoing HM compartmentalization [91]. Metals enter cells via cation transporters with a wide range of substrate specificity [91,92]. Overall, it is quite clear that CA can regulate HM stress directly through phytochelation and then store HMs in the vacuole, but very little is known about the movement of CA-HM complexes across the tonoplast membrane via vacuolar transporters. More research is needed to identify the role of the vacuolar transporters for CA-HM compartmentalization. Similarly, little is known about the release of CA from the HM chelation complex or its remobilization back outside the vacuole. 

### 4.2. Regulation of ROS and Antioxidants

Many recent studies have demonstrated that the application of exogenous CA can provide protection against oxidative stress in plants through increasing the activity of antioxidant defense systems [16,46,53,70,93]. Drought, flooding, heat, cold, salinity, and HM stress can lead to elevated ROS levels and result in disturbance of the cellular redox balance, leading to oxidative or nitrosative stress [94] and induction of antioxidant enzyme activities [95,96]. Oxidative stress results in cellular damage through membrane lipid peroxidation, natural antioxidant blockage, and reduced photosynthesis [97]. Antioxidant enzymes work to scavenge ROS and limit oxidative damage in the plant. CAT and APX directly detoxify ROS by converting H_2_O_2_ to water and oxygen [98], while SOD protects plants from oxidative damage by converting O_2_^•−^ (superoxide anion) to H_2_O_2_ [4,5].

The non-enzymatic and enzymatic components of the antioxidant defense system work together to scavenge ROS under stress conditions [5,98]. Build-up of CA due to redox-dependent inhibition of aconitase (ACO) during hypoxia has been suggested to induce metabolic changes as a stress adaptation strategy [99]. Plants react to stresses by activating the enzymatic defense system [100], a process facilitated by CA accumulation [17,18,43,59]. In several studies, both under non-stress and various stress conditions, the role of CA in promoting antioxidant enzyme activities has been reported [16,58,101,102,103]. CA functions as an elicitor of phenylpropanoid-derived compounds and activates signaling cascades to increase antioxidant activity [104]. Other interpretations of CA’s role in abiotic stress tolerance have been proposed as well. Zhao et al. [105] reported that CA functions as an antioxidant intermediate involving the defense pathways in response to abiotic stress. A similar study reported endogenous CA functioned primarily as an antioxidant and intermediate in respiration metabolism involving the defense pathways in response to high temperature stress [105]. 

Alternative oxidase (AOX) facilitates lower ROS levels by augmenting the capability of mitochondrial electron transport and inhibiting the production of O_2_^•−^ [106]. Importantly, the most powerful inducer of AOX expression yet reported is CA [107]. It is possible that higher endogenous CA, whether caused by metabolic engineering or exogenous application, will limit ROS-induced damages by promoting higher AOX activity (Figure 3). In support of this hypothesis, a recent study reported that ACO inhibition mediated by higher CA induced AOX activity in *Arabidopsis thaliana* under hypoxia and limited ROS production in mitochondria [99]. Moreover, a study on rice by Khatun et al. [7] reported that the activity of antioxidant enzymes (such as glutathione reductase, GR; GPX; SOD; CAT and glutathione *S*-transferase, GST) and antioxidant metabolites (such as GSH, proline, and carotenoid) increased significantly after CA supplementation, suggesting the active involvement of CA in ROS scavenging. CA can promote several enzymatic and non-enzymatic antioxidants and AOX activity and thereby help ameliorate damage by stress-induced ROS and enhance stress tolerance of plants (Figure 3).

### 4.3. Regulation of Osmoregulators and Secondary Metabolites

Plant cells accumulate osmolytes and SMs in part to protect cellular components from osmotic and oxidative stresses [108,109]. The most abundant osmolytes in plant cells are proline, glycine betaine, polyamines, and soluble sugars [109]. SMs including phenolics such as flavonoids, anthocyanins, and lignins [108] play roles in protecting plant cells from oxidative stress by scavenging free radicals [110,111]. There is insufficient evidence regarding the potential of CA to regulate the production of SMs under HM stress conditions, though at least three studies have reported an increase in SM synthesis, primarily flavonoids, after the application of exogenous CA. 

Plants experiencing environmental stress conditions accumulate proline in the leaves and proline levels correlate with stress tolerance [109]. In several studies, CA has been shown to stimulate synthesis of proline and other metabolites (including phenolic compounds, flavonoids, tannins, and sugars) in plants experiencing abiotic stress conditions [68,112,112,113]. In *Arabidopsis thaliana*, CA enhances the biosynthesis of amino acids such as proline, glycine, serine, leucine, and lysine [99]. HMs upset the water balance in plants and lower water potential [68]. Proline stabilizes subcellular structures and molecules experiencing osmotic stress conditions by working as a molecular chaperone, maintaining the integrity of proteins [114,115]. It also serves as an antioxidant in its own right [114,115]. Exogenous application of CA led to increased proline content in *B. juncea* grown under Cd stress [61,68], thereby protecting against HM stress. 

Phenolic compounds accumulate in cellular vacuoles through hydrolyzation and decomposition of cellular components and cell walls [116,117]. Limón et al. [118] and Li et al. [113] reported that CA increased cellular phenolic compounds by eliciting the degradation of polyphenols (e.g., tannins) into simple phenols. Such phenols may have protective benefits for plants under HM or other osmotic- or oxidative-stress inducing conditions.

Treatment with CA has also been shown to promote anthocyanin and flavonoid accumulation [68,101]. A probable mechanism for this relationship was identified in Cd-stressed *B. juncea* plants where exogenous CA treatment enhanced chalcone synthase (*CHS*) gene expression [68]. Another study showed that in wheat sprouts treated with CA, signal transduction pathways leading to increased secondary metabolites accumulation were activated [101]. Exogenous CA lowered the pH which enhanced the release of flavonoids and anthocyanins [119]. 

## 5. Genetic Engineering for CA-Mediated Abiotic Stress Tolerance

Genetic engineering offers a promising approach to modulate CA metabolism in plants for improved abiotic stress tolerance (Table 5). A favored approach has been to increase CA biosynthesis by overexpressing CA biosynthetic genes like CS, which converts OAA and acetyl-CoA to CA during the TCA cycle [6], or PEPC, that produces OAA from PEP [8]. Several studies have demonstrated the utility of overexpressing CS-encoding genes in overcoming Al stress. Transgenic tobacco, papaya, and Arabidopsis overexpressing CS from *Pseudomonas aeruginosa* showed higher tolerance to Al-toxicity [11,120,121,122]. Likewise, overexpression of CS from *Malus xiaojinensis* in tobacco led to higher CA content and improved tolerance to Fe-stress [123]. However, overexpression alone is not always sufficient to cause increased CA accumulation or HM tolerance [124]. Transgenics overexpressing mitochondrial isoforms of CS (*mtCS*) have also been employed. Koyama et al. [121] overexpressed carrot *mtCS* in Arabidopsis and showed a 60% increase in CA efflux and better performance under toxic Al concentrations. Similar results were obtained in transgenic canola overexpressing Arabidopsis *mtCS*, where increased CA exudation from roots was shown to directly correlate with transgene expression [125]. Importantly, CS encompasses only a small part of the complex system behind CA metabolism and genetic manipulation of several metabolite enzymes at once (super expression strategies), such as malate dehydrogenase (MDH), CS, and PEPC, may increase the synthesis and accumulation of CA even more [125]. A contrasting strategy has been to down-regulate CA catabolism by repressing ACO and isocitrate dehydrogenase (IDH) using an antisense approach, and thus increase CA concentration and efflux from roots [125,126]. 

Another popular target for genetic manipulation has been anion channels in the plasma membrane that play a major regulatory role in the transport of CA from roots [127]. Transporters for CA anions include members of the Al-activated malate transporter (ALMT) and multidrug and toxic compound extrusion (MATE) families [128,129]. The *FeMATE1* involved in the Al-induced secretion of citrate in the roots, while *FeMATE2* transports citrate into the Golgi system for internal detoxification of Al in both the roots and leaves of *Fagopyrum esculentum* [130]. In ricebean (*Vigna umbellata*) grown under Al toxicity, *VuMATE2* and *VuMATE1* control the CA efflux from roots in the early phase and late phase growth, respectively [131]. MATE transporters underlying aluminum-activated CA secretions have been identified in various species, including *ZmMATE1* (*Zea mays*) [132], *ScFRDL2* (*Secale cereale*) [133], *OsFRDL4* (*Oryza sativa*) [134], *AhMATE1* (*Amaranthus hypochondriacus*) [135], and *HvAACT1 (Hordium vulgare)* [136]. Two genes, *BdMATE* and *SbMATE* obtained from *Brachypodium distachyon* and *Sorghum bicolor*, respectively, were overexpressed in *Setaria viridis* and caused increased CA secretion from the apex of the root [137,138].

Lastly, the role of CA in alkaline stress tolerance has also been a target of genetic engineering. Zhu et al. [139] showed improved alkaline tolerance in transgenic *M. sativa* overexpressing *TIFY10a* gene from *Glycine soja*. These findings suggested that the ability to maintain cytosolic pH homeostasis through increased NADP-ME (NADP-dependent malic enzyme) activity and CA content could alleviate high pH damage. Results from Sun et al. [140] revealed that transgenic *M. sativa* produced through the overexpression of the *Glycine soja* PEPC kinase 3 (*PPCK3*) gene exhibited higher levels of CA and performed better under alkali stress. 

Genetic engineering for enhanced CA accumulation can improve Al, Fe, and alkalinity stress tolerance. The identification of genes regulating CA synthesis and transport, the determination of their expression patterns in response to stress, and a deeper understanding of their functions in stress adaptation will further enable genetic engineering and breeding technologies for improved stress tolerance. Evidence for whether CA over-accumulation can promote resiliency against other stresses is lacking and further studies are needed.

## 6. Metabolism of CA and Its Role in the Biosynthesis of Secondary Metabolites, Signaling Molecules, and Phytohormones

**Figure 4:**
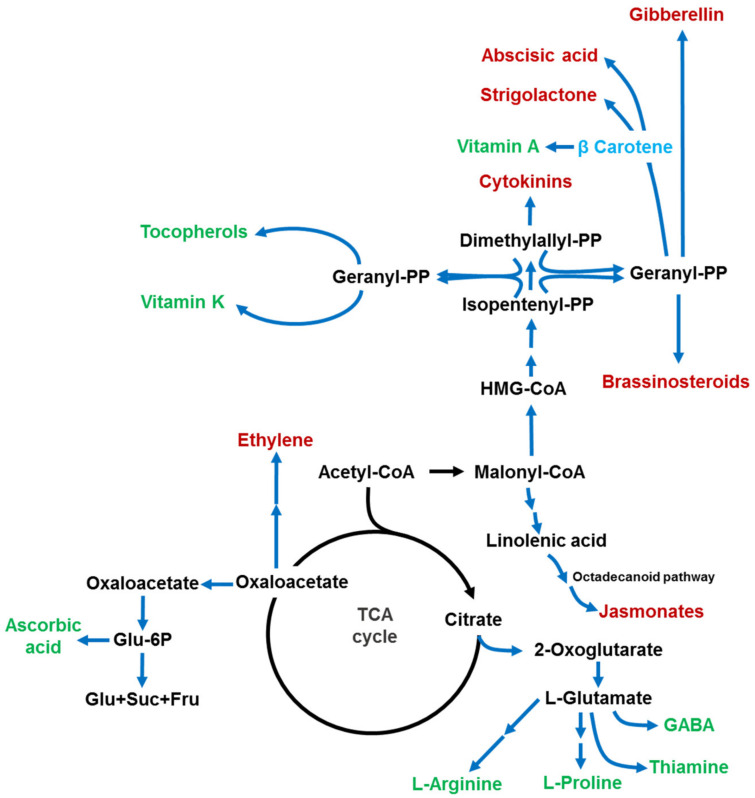
Schematic representation of CA metabolism in plants. Citrate derived from the TCA cycle can be converted to acetyl-CoA. Acetyl-CoA carboxylase converts acetyl-CoA to malonyl-CoA, a precursor for fatty acid and jasmonate biosynthesis via the octadecanoid metabolic pathway. Malonyl Co-A also feeds into the mevalonate pathway and provides building blocks of phytohormones (cytokinins, gibberellins, abscisic acid, brassinosteroids, and strigolactones) and vitamins (vitamin K and vitamin A). Oxaloacetate (OAA) can be converted into glucose-6-phosphate via PEP caroxykinase and phosphatases, providing a source of ascorbic acid as well as glucose, sucrose and fructose. 2-oxoglutarate can be converted into glutamate, feeding into GABA and amino acid biosynthesis.

Citric acid, the 1st intermediate of the TCA (Krebs) cycle, is central to numerous interrelated metabolic networks that produce a myriad of SMs, amino acids, phytohormones and OAs [6,143,144], many of which play roles in abiotic stress tolerance in plants [145]. During the TCA cycle, the condensation of OAA and acetyl-CoA yields CA [146,147], which can then be utilized for the biosynthesis of γ-aminobutyric acid (GABA) [148], isoprenoids, flavonoids, fatty acids, sugars, and hormones (Figure 4) [149].

Citric acid can be utilized for amino acid or GABA biosynthesis through the production of glutamate [148,150]. ACO, IDH, glutamate synthase (GS), and glutamate decarboxylase (GAD) are key enzymes involved in CA catabolism through the ACO-GABA pathway [9]. The citric acid cycle intermediates α-ketoglutarate (α-KG) and 2-oxoglutarate (2-OG) feed into the ACO-GABA pathway [151]. An oxidative deamination process converts α-KG to glutamate via glutamate dehydrogenase [152]. This glutamate can be utilized by two alternative pathways, one involving the conversion of glutamate into glutamine and the other processing glutamate through the GABA shunt [8,9,148,150,153]. Glutamate serves as a precursor for many amino acids and amino acid-derived compounds including proline, arginine, ornithine, thiamine, and lysine [154]. Elevated levels of CA in the cytosol have been shown to enhance the activity of enzymes of the GABA shunt pathway in *Citrus limon* callus and citrus fruits [8,150].

Another destination for CA is the acetyl-CoA pathway, alternatively known as the ATP citrate lyase (ACL) pathway [151,155,156], which utilizes CA for biosynthesis of numerous secondary metabolites via either the mevalonate (MVA) pathway or the non-MVA pathway [9,151,157]. Acetyl-CoA produced from CA is primarily utilized for the biosynthesis of isoprenoids and other SMs [149,158,159]. The mevalonate (MVA) pathway uses acetyl-CoA to synthesize the universal isoprenoid precursor isopentenyl diphosphate, a substrate for the biosynthesis of many important metabolites and phytohormones including GA, carotenoids, abscisic acid (ABA), strigolactones, cytokinins (CK), brassinosteroids (BR), and tocopherols (Figure 4) [160,161,162]. In addition, acetyl-CoA is utilized for fatty acid elongation which ultimately can lead to jasmonate biosynthesis through the non-MVA octadecanoid pathway (Figure 4) [163]. 

Finally, OAA, another product of ACL catalysis and intermediate in the citric acid cycle, can be utilized for gluconeogenesis to produce soluble sugars (sucrose, fructose, and glucose) and for the synthesis of organic acids such as ascorbate and aspartic acid (Figure 4) [9,154]. Furthermore, OAA is needed for ethylene biosynthesis in plants [151].

In summary, CA and other CA-derived TCA cycle intermediates are intimately involved in the complex metabolic networks leading to the biosynthesis of many phytohormones, amino acids, SMs, and OAs. Many of these compounds play roles in amelioration of abiotic stresses including drought, salinity, light, temperature, air pollution, and HM toxicity [144]. As discussed previously, amino acids including GABA, proline, arginine, glutamine, and aspartic acid have been shown to contribute to osmotic and oxidative stresses. Similarly, the major classes of phytohormones and related metabolites including BR, GA, ABA, CK, carotenoid, strigolactones, ethylene, tocopherols, and thiamine have been shown to play important roles in abiotic stress tolerance. Moreover, soluble sugars like sucrose, fructose, glucose and glucose-6-phosphate-derived from ascorbic acid (AsA) also contribute to abiotic stress adaptation. Therefore, the role of CA in abiotic stress tolerance is complex and likely involves the biosynthesis of stress-mitigating phytohormones, SMs, OAs, and sugars.

Several studies have corroborated the hypothesis that stress ameliorating effect of CA may involve its position in secondary metabolism. For example, the GABA pathway is associated with temperature stress response in *Citrus sinensis* (blood orange) [158,164]. Hot air treatment of mandarin fruits led to degradation of organic acids including CA and the accumulation of soluble sugars, a response involving the ACO-IDH-GAD cascade [165]. Alternatively, the ACL pathway utilizing CA for flavonoid biosynthesis is associated with stress mitigation in cold-stressed blood oranges [164]. Additionally, overexpression of *ACLA-1* gene of *Saccharum officinarum*, associated with CA catabolism in the ACL pathway, enhanced drought tolerance in tobacco [166]. Exogenous CA application ameliorated Cd stress in *B. napus*, a response associated with higher total soluble sugars, Chl, and carotenoid contents [16]. Crosstalk amongst stress-ameliorating OAs, SMs, phytohormones, and CA likely contributes to CA-mediated stress tolerance as suggested by Ye et al. [167] and Sadak et al. [168], each of whom showed increased levels of GA, BRs, indole acetic acid (IAA), and decreased ABA content after AsA and CA treatment. However, more research is needed to clarify the complex mechanisms and interactions between CA-dependent metabolites and their individual and combined influences on stress tolerance in plants.

## 7. Conclusions and Future Perspective

It can be concluded from the above discussion that exogenous CA application by foliar sprays or through rooting medium can effectively modulate various plant growth responses under diverse environmental stress conditions. In sum, exogenous CA:

- Enhances growth, photosynthesis, and many physio-biochemical parameters that promote crop productivity under abiotic stress conditions.
- Alleviates the abiotic stress-induced osmotic imbalance by increasing osmoregulators and protecting membranes from damage.
- Reduces the severity of oxidative stress by upregulating non-enzymatic and enzymatic antioxidants.
- Accelerates the HM stress tolerance of plants by chelating and sequestering HMs and improves HM phytoextraction from HM-polluted soils.
- Provides the substrate for a wide variety of metabolic pathways synthesizing stress protectant metabolites including phytohormones, amino acids, organic acids, and fatty acids.

Still, the role of CA in abiotic stress tolerance has not yet been studied exhaustively and additional research is needed. A number of important relationships between CA and stress responses have not been examined. For example, the role of CA in cold stress tolerance has not been reported. The influence of CA on plant growth regulators, such as auxins, ABA, CK, GA, ethylene, salicylic acid, jasmonates, BRs, etc. are largely unknown. The interaction between exogenous CA and AOX has not been shown. Moreover, malate, an OA structurally similar to CA, can regulate anion channels in guard cells and involved in stomatal signaling [169], but the role of CA in stomatal signaling remains unknown. Finally, the effect of CA application on plant defense genes and interactions with biotic stresses remains largely unexplored. With the development of advanced omics technologies, more detailed research will emerge that explores CA-mediated stress tolerance at the transcriptome, proteome, and metabolome levels.

The use of biostimulants and chemical protectants has potential to overcome abiotic stress-caused losses on crops yields [170]. Many reports have shown that exogenous application of naturally-occurring plant chemicals such as salicylic acid, hydrogen peroxide, calcium, glutathione, ABA, jasmonic acid, polyphosphoinositides, nitric oxide, thiourea, and others can mitigate various abiotic stresses [4,171,172,173,174,175].

CA is a weak OA that occurs naturally in plants and to particularly high levels in citrus fruits. It is generally recognized as safe by the Food and Drug Administration and has no associated health concerns. CA is inexpensive to synthesize and to apply exogenously to crops, most cost-effectively by foliar spray along with post-emergent herbicides, insecticides, or fungicides. Exogenous application of CA is a promising low-cost approach to help alleviate abiotic stresses and promote crop yield.

## Figures and Tables

**Table 1:** Published effects of abiotic stresses on endogenous CA levels in plants.

Stress	Treatment	Plant Species	Organ/Tissue	Duration	Endogenous CA Level	Reference
Salinity	50 to 250 mM NaCl	*Helianthus annuus*	Shoot	7 days	↑	[19]
	20 and 120 mM NaCl	*Solanum lycopersicum*	Shoot	10 days	↑	[21]
	100 and 200 mM NaCl		Root exudates	4 weeks	↑	[20]
	100 and 200 mM NaCl	*Acacia nilotica*	Root exudates	4 weeks	↑	[20]
	25 to 200 mM NaCl	*Trigonella foenum-graecum*	Seedling	5 days	↑	[22]
Drought	40, 70, and 100% FC	*Solanum lycopersicum*	Fruit	120 days	↑	[27]
	Irrigated and dryland	*Gossypium hirsutum*	Leaf	108 days	↑	[23]
	Withholding water	*Clusia* sp.	Leaf	16 days	↑	[24]
	Withholding water	*Aptenia cordifolia*	Leaf	10 days	↑	[25]
	−20, −20 to −40, and −40 to −60 kPa	*Solanum tuberosum*	Tuber	42 days	=	[26]
Heat	25/20 °C and 35/30 °C (D/N)	*Festuca arundinacea*	Leaf	28 days	↑	[29]
	22°C and 30 °C (daytime)	*Solanum tuberosum*	Tuber	42 days	=	[26]
	20/15 °C and 35/30 °C	*Poa pratensis*	Leaf	18 days	=	[28]
	30/25 °C and 45/40 °C	Hybrid bermudagrass	Leaf	18 days	↑	[28]
	25/20 °C and 35/30 °C (D/N)	*Lolium arundinaceum*	Leaf	15 days	↑	[17]
HMs	50 µM CdCl_2_	*Solanum nigrum*	Root	24 h	↑	[30]
	0.6 mM CdCl_2_	*Brassica juncea*	Shoot	7 days	↑	[33]
	150 μM NiCl_2_.6H_2_0	*Amaranthus paniculatus*	Leaf and root	1 week	↑	[32]
	50 µM K_2_Cr_2_O_7_	*Oryza sativa*	Root exudates	8 days	↑	[34]
	100 µM K_2_Cr_2_O_7_	*Oryza sativa*	Root exudates	8 days	↓	[34]
	100 µM K_2_Cr_2_O_7_	*Oryza sativa*	Root exudates	16 days	↑	[34]
	50 μM AlCl_3_	*Secale cereale* and *Triticum aestivum*	Root exudates	12 h	↑	[37]
	15 μM AlCl_3_.6H_2_O	*Glycine max*	Root exudates	24 h	↑	[39]
	50 μM AlCl_3_	*Brachiaria brizantha*	Root exudates	12 h	↓	[36]
	30 μM AlCl_3_	*Cassia tora*	Root exudates	9 h	↑	[35]

↑ CA increase; ↓ CA decrease; = CA unchanged; FC, Field Capacity; D/N, day/night.

**Table 2:** Effectiveness of exogenous CA on mediating salinity and drought stress tolerance.

Plant Species	Stress	CA Treatments and Method of Application	Effects	Outcomes	References
*Gossypium barbadense* (Cotton)	Salt (205, 135, and 35 mM NaCl)	Foliar spray of 2.5 g L^−1^ potassium citrate	Increased growth, yield, and photosynthetic pigments. Increased TSS, TSP, TPC, FAA, and proline. Enhanced CAT, POX, and SOD activities.	Improved growth and yield but no effects on fiber properties. Increased salt tolerance.	[18]
*Carica papaya* (Papaya)	Salt (NaCl)	Seed soaking with CA (500 mg L^−1^) as sildenafil citrate	Increased germination rate.	Improved the tolerance and development of papaya plants in saline environments.	[42]
*Phaseolus vulgaris* (Common Bean)	Drought	Spraying of CA (0.5, 1.0, 1.5, and 2 g L^−1^)	Increased relative water content (RWC) and Chl. Increased plant growth and productivity.	Application of CA at 1.5 g L^−1^ was most effective for drought alleviation.	[14]
*Zea mays* (Maize)	Salt (NaCl) (4.2–4.6 dSm^−1^)	Foliar spray of CA with ascorbic acid and salicylic acid (100 or 200 ppm)	Increased leaf area index, net assimilation rate, growth rate, and photosynthetic pigments. Enhanced CAT, POX, PPO, and PAL activities. Decreased proline and Na^+^. Increased K^+^.	Improved tolerance to salinity. Enhanced growth and yield.	[43]
*Leymus chinensis* (Chinese ryegrass)	Salt (200 mM NaCl) and alkaline stress (100 mM Na_2_CO_3_)	Irrigation with CA (50 mg L^−1^)	Increased growth and CA exudation. Increased RWC and CO_2_ assimilation rate. Enhanced MDA content, CAT, APX, and SOD activities.	Improved tolerance to saline and alkaline stress.	[46]
*Gossypium barbadense* (Cotton)	Drought	Foliar spray of CA (500 ppm)	Increased growth, number of fruiting branches, number of open bolls per plant, seed index, boll weight, lint percentage, and seed cotton yield. Increased Chl *a*, Chl *b*, Chl *a*+*b*, carotenoid, and proline contents in leaves. Enhanced CAT and POX activities.	Reduced drought sensitivity but no significant effects on fiber properties.	[15]
*Hibiscus sabdariffa* (Roselle)	Salt (75 mM NaCl)	Foliar spray of CA (10 mM)	Increased TPC and proline accumulation. Reduced GSH content. Enhanced SOD activity but decreased CAT, POX, and PAL activities	Improved flower production under salinity condition.	[45]
*Brassica oleracea* (Cabbage)	Drought	Spraying of CA (5 mM)	Increased P uptake. Decreased hydrogen peroxide production.	Alleviated drought-induced oxidative stress.	[49]
*Melissa officinalis* (Lemon balm)	Salt (0.0, 1.6, 3.1, and 6.3 dSm^−1^)	Foliar spray of CA (0.3 g L^−1^)	Increased levels of α-pinene, β-bisabolene, monoterpene hydrocarbons (MCH) and oxygenated sesquiterpenes (SCHO).	Improved growth.	[44]
*Beta vulgaris* (Sugar beet)	Salt (12.50 dSm^−1^)	Soil application of CA (300 mg L^−1^)	Increased K, N, and P when added in combination with tomato peel extract. Increased CAT and POX activity when added in combination with banana peel extract.	Banana extract and CA reduced soil salinity. Increased root and sugar yield.	[47]

**Table 3:** Effectiveness of exogenous CA on mediating temperature and alkaline stress tolerance.

Plant Species	Stress	CA dose	Effects	Outcomes	Reference
*Lolium arundinaceum*	Heat stress: (25/20 °C and 35/30 °C, day/night) in growth chambers	Foliar spraying of CA (0, 0.2, 2, and 20 mM)	Increased growth. Increased Chl content, photochemical efficiency (Fv/Fm) and SOD, POX, and CAT activities. Decreased EL and MDA content. Increased expression of heat shock protein genes.	Alleviated growth and physiological damage caused by high temperature	[17]
*Lycopersicon esculentum*	Heat stress as manipulated by late summer sowing (air temp up to 35 °C)	Spraying of CA (2.5 and 5 g L^−1^)	Increased yield and fertility of pollen grains. Increased vitamin C content, TSS, minerals. Increased stem thickness, epidermis, phloem and xylem tissues. Enhanced POX, SOD, and CAT activities.	Increased yield during late summer	[50]
*Hibiscus rosa-sinensis*	Cold stress (˂10 °C)	CA (5 mM) in nutrient solution	Increased the number of leaves remaining on plants grown under low-illumination.	Suppressed defoliation	[51]
*Leymus chinensis*	Alkaline stress (100 mM Na_2_CO_3_)	Spraying of CA (50 mg L^−1^)	Increased growth, relative growth rate, and photosynthesis. Enhanced CAT, SOD, and APX activities.	Increased stress tolerance	[46]
*Rosa roxburghii*	Calcareous yellow soil (pH higher than 8)	CA (40, 80 and 120 mg kg^−1^ soil)	Increased growth, total biomass, root development, root-shoot ratio, and total root surface area. Increased nutrient contents.	Increased seedling growth	[52]

**Table 4:** Effectiveness of exogenous CA on mediating HM stress tolerance.

Plant Species	HM Stress	Treatments	Effects	Outcomes	References
*Brassica napus*	Cu (50 and 100 µM as CuSO_4_)	CA (2.5 mM) in nutrient solution	Increased plant growth, biomass, Chl content, stomatal conductance, and water use efficiency. Enhanced POX, SOD, CAT, and APX activities. Reduced H_2_O_2_, MDA, and EL.	Minimized Cu toxicity and enhanced biomass production.	[56]
*Brassica napus*	Cd (10 and 50 µM as CdCl_2_)	CA (2.5 mM) in solution medium	Enhanced plant growth and biomass, gas exchange activities, and antioxidant enzymes activity. Reduced oxidative stress by reducing H_2_O_2_ and MDA production and decreasing EL.	Mitigated Cd stress.	[16]
*Solanum nigram*	Cd (50 mg Cd^2+^ kg^−1^ dry soil)	CA (20 mmol kg^−1^ soil) applied in soil	Promoted plant growth, biomass, and antioxidative defense e.g., SOD and POX activity at initial stage.	Slightly reduced Cd stress.	[70]
*Brassica juncea*	Cd (0.6 mmol kg^−1^ soil as CdCl_2_)	CA (0.6 mmol kg^−1^ soil) applied in soil	Increased plant height, Chl *a*+*b*, carotenoid, anthocyanins, and flavonoids in leaves. Non-significant increment of the activities of SOD, POX, CAT, and GPX. Reduced MDA levels.	Alleviated Cd-induced toxicity.	[68]
*Brassica juncea*	Cd (0.6 mM) as CdCl_2_	Soil treatment with CA (0 and 0.6 mM)	Significantly increased Chl *a*+*b*, carotenoid, and polyphenols. Non-significant increase in flavonoids, anthocyanins and total carbohydrate content. Induced stomatal opening. Reduced ROS production.	Alleviated Cd stress.	[33]
*Brassica napus*	Cr (100 and 500 μM)	Irrigated with CA (2.5 and 5.0 mM)	Increased plant growth, biomass, Chl *a*, Chl *b*, Chl *a*+*b*, carotenoid, and soluble protein concentrations. Enhanced activities SOD, POX, CAT, and APX. Reduced MDA and EL.	Improved Cr stress tolerance.	[58]
*Brassica juncea*	Cd (0.5 mM Cd and 1.0 mM CdCl_2_)	CA (0.5 and 1.0 mM) in nutrient solution	Increased plant growth, leaf RWC, and Chl content. Enhanced activities of APX, MDHAR, DHAR, GR, GPX, SOD, and CAT. Reduced oxidative damage.	Enhanced Cd stress tolerance by regulating antioxidant defense.	[53]
*Helianthus annuus* (Sunflower)	Cr (5, 10 and 20 mg kg^−1^ dry weight)	CA treatment (2.5 and 5.0 mM)	Increased plant growth and biomass, Chl, carotenoid, photosynthesis, gas exchange, and soluble proteins. Enhanced activities of antioxidant enzymes. Reduced production of ROS and MDA.	Improved Cr stress tolerance.	[59]
*Juncus effusus*	Mn (50, 100 and 500 μM as MnSO_4_)	CA (5 mM) in the nutrient solution	Increased shoot length and root number.	Alleviated Mn toxicity and enhanced growth.	[71]
Germinating pea seeds	Cu (as 200 µM CuCl_2_)	Irrigated with CA (as 100 µM Na-citrate)	Reduced oxidative stress. Decreased H_2_O_2_, MDA, carbonyl groups, lipid peroxidation, and protein oxidation.	Enhanced growth and reduced stress.	[67]
*Zea mays* (Maize)	Cd as CdCl_2_ (300 mg kg^−1^)	Irrigation with CA (0.25, 0.5, 1.0 and 2 g kg^−1^ soil)	Increased root and shoot length, biomass. Reduced bioaccumulation coefficient and translocation factor. Reduced Cd uptake.	CA proved inefficient for Cd phytoextraction, however, ameliorated the toxicity of Cd	[63]
*Brassica juncea*	Cd (150 mg Cd^2+^ kg^−1^ soil)	CA (10 and 20 mmol kg^−1^ soil)	Increased shoot phenolic acids. Reduced ROS production.	Improved Cd stress tolerance.	[66]
*Brassica napus*	Pb as Pb(NO_3_)_2_ (50 and 100 μM)	CA (2.5 mM) in solution media	Increased plant height, root length, leaf growth, fresh and dry weight, Chl content, SPAD values, Pn, E, Gs, and Pn/E. Enhanced SOD, POX, CAT, and APX activities. Prevented lipid membrane damage. Reduced MDA and H_2_O_2_ production.	Increased Pb stress tolerance.	[57]
*Solanum lycopersicum*	Pb (10 μM as Pb(NO_3_)_2_) and As (10 μM as Na_2_HAsO_4_)	CA (250 μM) in nutrient solution	Increased Chl *a* and Chl *b* content. Decreased Pb accumulation, α-tocopherol content, and MDA levels.	Increased Pb and As tolerance.	[69]
Roots of *Vicia faba*	Pb (5 μM) as Pb(NO_3_)_2_	CA (550 μM and 1000 μM) in nutrient culture	Non-significant effect on antioxidant enzyme activities (i.e., SOD, GPX, APX, and GR).	CA did not mitigate Pb toxicity	[73]
*Sedum alfredii*	Cd (100 µmol L^−1^ CdCl_2_)	CA (0, 10, 50, 100, 500 µmol L^−1^) in solution culture	Increased plant growth and biomass.	Improved Cd stress tolerance	[74]
*Corchorus olitorius*	Cd (20 mg L^−1^) as Cd(NO_3_)_2_. 4H_2_O	5 mM CA in nutrient culture	Enhanced antioxidant enzyme activity. Decreased Cd^2+^ uptake and accumulation.	Improved Cd stress tolerance	[62]
*Salix variegate*	Cd (50 μmol L^−1^) as CdCl_2_·2. 5H_2_O	CA (100 μmol L^−1^) in nutrient solution	Increased biomass, carotenoid, Chl *a*, Chl *b* and Chl *a*+*b* content. Increased net photosynthesis rate, stomatal conductance, chloroplast size and width.	Reduced stress and enhanced growth, biomass, and photosynthesis.	[55]
*Brassica juncea*	Ni as NiSO_4_ (0.003 mmol L^−1^)	CA (0.5, 1.0, and 5.0 mmol L^−1^) in nutrient solution	Reduced Ni uptake but had no effect on Ni translocation.	Reduce stress by reducing Ni uptake.	[65]
*Brassica juncea*	Cd (0.6 mM)	Foliar spray of CA (0.6 mM)	Increased plant growth. Increased antioxidant activity. Reduced ROS.	Enhanced growth and efficacy of photosynthetic machinery	[61]
*Helianthus annuus* (Sunflower)	Cr (5, 10, and 20 mg kg^−1^)	Irrigation with CA (2.5 and 5 mM)	Increased plant growth, Chl, carotenoid, Pn, E, Gs, and water use efficiency.	Increased tolerance to Cr stress.	[40]
*Larix olgensis*	100 mg kg^−1^ Pb from Pb(NO_3_)_2_	Root irrigation and foliar spraying of CA (0.2, 1.0, 5.0, and 10.0 mmol L^−1^)	Increased plant growth and biomass, proline, total Chl, and carotenoid content. Enhanced SOD and POX activities. Reduced Pb content and MDA levels.	Improved tolerance to Pb stress	[54]
*Oryza sativa* (Rice)	Cd as CdCl_2_ (25.0 µM)	CA (50.0 µM) in nutrient solution	Increased GSH, Chl, carotenoid, and anthocyanin contents. Decreased Cd content in leaves.	Enhanced Cd tolerance and promoted higher biomass production	[60]
*Triticum aestivum* (Wheat)	20 µM Cd (added as CdCl_2_)	Irrigation with CA (10, 50, 100, and 500 µM)	Increased index of tolerance, root and shoot biomass. Decreased Cd uptake, MDA levels, and PCs-SH production in roots.	Reduced bioavailability of Cd.	[64]
*Medicago sativa* (Alfalfa)	100 µM Al in nutrient solution	Foliar spraying with 100 µM of CA	Increased growth. Reduced lipid peroxidation.	Alleviated Al toxicity through roots Al detoxification	[75]
*Typha latifolia*	Pb and Hg (1, 2.5 and 5 mM)	CA (5 mM) in nutrient medium	Increased fresh and dry biomass of root, stem, and leaf. Increased Chl *a*, Chl *b*, Chl *a+b*, carotenoid, soluble protein contents, and SPAD values. Decreased ROS, MDA, and EL. Enhanced the activities of SOD, POX, APX, and CAT.	Improved stress tolerance with increased physiological parameters.	[72]

**Table 5:** Examples of transgenic plants overexpressing genes for CA biosynthesis and their phenotypic response to abiotic stresses.

Gene(s)	Origins	Transgenic Plants	Phenotype	References
Citrate Synthase (*CS*)	*Pseudomonas aeruginosa*	*Nicotiana tabacum*	Al stress tolerance	[120]
*CS*	*Pseudomonas aeruginosa*	*Papaya* sp.	Al stress tolerance	[120]
*CS*	*Pseudomonas aeruginosa*	Tobacco plant	Al stress intolerance	[124]
*AACT1*	*Hordeum vulgare*	Tobacco cells	Al stress tolerance	[136]
*MATE*	*Sorghum bicolor*	*Arabidopsis thaliana*	Al stress tolerance	[137]
*CS*	*Pseudomonas aeruginosa*	*Medicago sativa*	Al stress tolerance	[141]
*CS*	*Citrus junos*	*Nicotiana benthamiana*	Al stress tolerance	[142]
*MATE1*	*Zea mays*	*Arabidopsis thaliana*	Al stress tolerance	[132]
*MATE*	*Vigna umbellate*	*Solanum lycopersicum*	Al stress tolerance	[134]
*MATE*	*Brachypodium distachyon*	*Setaria viridis*	Al stress tolerance	[138]
Mitochondrial Citrate Synthase (*mtCS*)	*Arabidopsis thaliana*	*Daucus carota*	Al stress tolerance	[121]
*mtCS*	*Arabidopsis thaliana*	*Brassica napus*	Al stress tolerance	[125]
*TIFY10a*	*Glycine soja*	*Medicago sativa*	Alkaline stress tolerance	[139]
*PPCK3*	*Glycine soja*	*Medicago sativa*	Alkaline stress tolerance	[140]
*CS1*	*Malus xiaojinensis*	*Nicotiana tabacum*	Fe stress tolerance	[123]

## Data Availability

Not applicable.
